# Yellow fever control in Cameroon: Where are we now and where are we going?

**DOI:** 10.1186/1741-7015-6-3

**Published:** 2008-02-08

**Authors:** Charles Shey Wiysonge, Emmanuel Nomo, Jeanne Mawo, James Ofal, Julienne Mimbouga, Johnson Ticha, Peter M Ndumbe

**Affiliations:** 1 Central Technical Group, Expanded Programme on Immunisation, Ministry of Public Health, Yaoundé, Cameroon; 2 South African Cochrane Centre, Medical Research Council, Cape Town, South Africa; 3 World Health Organization, Yaoundé, Cameroon; 4 Faculty of Health Sciences, University of Buea, Buea, Cameroon

## Abstract

**Background:**

Cameroon is one of 12 African countries that bear most of the global burden of yellow fever. In 2002 the country developed a five-year strategic plan for yellow fever control, which included strategies for prevention as well as rapid detection and response to outbreaks when they occur. We have used data collected by the national Expanded Programme on Immunisation to assess the progress made and challenges faced during the first four years of implementing the plan.

**Methods:**

In January 2003, case-based surveillance of suspected yellow fever cases was instituted in the whole country. A year later, yellow fever immunisation at nine months of age (the same age as routine measles immunisation) was introduced. Supplementary immunisation activities (SIAs), both preventive and in response to outbreaks, also formed an integral part of the yellow fever control plan. Each level of the national health system makes a synthesis of its activities and sends this to the next higher level at defined regular intervals; monthly for routine data and daily for SIAs.

**Results:**

From 2004 to 2006 the national routine yellow fever vaccination coverage rose from 58.7% to 72.2%. In addition, the country achieved parity between yellow fever and measles vaccination coverage in 2005 and has since maintained this performance level. The number of suspected yellow fever cases in the country increased from 156 in 2003 to 859 in 2006, and the proportion of districts that reported at least one suspected yellow fever case per year increased from 31.4% to 68.2%, respectively. Blood specimens were collected from all suspected cases (within 14 days of onset of symptoms) and tested at a central laboratory for yellow fever IgM antibodies; leading to confirmation of yellow fever outbreaks in the health districts of Bafia, Méri and Ntui in 2003, Ngaoundéré Rural in 2004, Yoko in 2005 and Messamena in 2006. Owing to constraints in rapidly mobilising the necessary resources, reactive SIAs were only conducted in Bafia and Méri several months after confirmation of the outbreak. In both districts, a total of 60,083 people (representing 88.2% of the 68,103 targeted) were vaccinated. Owing to the same constraints, SIAs were not conducted promptly in response to the outbreaks in Ntui, Ngaoundéré Rural, Yoko and Messamena. However, these four and two other health districts at high risk of yellow fever outbreaks (i.e. Maroua Urban and Ngaoundéré Urban) conducted preventive SIAs in November 2006, vaccinating a total of 752,195 people (92.8% of target population). In both the reactive and preventive SIAs, the mean wastage rates for vaccines and injection material were less than 5% and there was no report of a serious adverse event following immunisation.

**Conclusion:**

Amidst other competing health priorities, over the past four years Cameroon has successfully planned and implemented evidence-based strategies for preventing yellow fever outbreaks and for detecting and responding to the outbreaks when they occur. In order to sustain these initial successes, the country will have to attain and sustain high routine vaccination coverage in each successive birth cohort in every district. This would require fostering and sustaining high-level political commitment, improving the planning and monitoring of immunisation services at all levels, adequate community mobilisation, and efficient coordination of current and future immunisation partners.

## Background

Yellow fever, which has caused devastating epidemics for centuries [1-3], continues to cause severe morbidity and mortality in Africa [4-6] despite the availability of an effective vaccine for the past 70 years [7]. The viral haemorrhagic disease is propagated in Africa by infected mosquitoes [8]. *Aedes aegypti *is the intermediary in the urban human-to-human cycle of transmission and various *Aedes *species are intermediaries in the jungle monkey-to-monkey cycle, with inadvertent human insertion into that cycle. The World Health Organization (WHO) estimates that the disease causes 200,000 cases and up to 30,000 deaths each year in the continent [9]. Most of these cases and deaths occur in 12 countries, including Cameroon [10]. Cameroon is situated in central Africa at the end of the Gulf of Guinea, between latitudes 2° and 13° north of the Equator and longitudes 9° and 16° east of the Greenwich Meridian. The country, whose current population is estimated at 18 million inhabitants of whom 46% live in urban areas, conducted periodic mass preventive yellow fever vaccination campaigns for about three decades beginning in the early 1930s [11]. These regular mass campaigns led to a rapid reduction in susceptible individuals and, consequently, the yellow fever burden in the country. Within two decades of cessation of the preventive vaccination campaigns, Cameroon started experiencing serious yellow fever epidemics [12,13], most of which remained unknown [14] owing to a weak disease surveillance system. In September-December 1990 during one epidemic in the Far North Province of the country, 182 cases (with 69% case-fatality rate) were reported [13], but it is estimated that up to 20,000 cases may have occurred during that epidemic [15]. Building upon experience gained in conducting high-quality polio eradication and measles elimination activities [16-19], in 2002 Cameroon developed a five-year (2003–2007) strategic plan for yellow fever control. In this report, we present progress and challenges of implementing the plan during its first four years of existence.

## Methods

The Cameroon government (through the Ministry of Public Health) planned and implemented the yellow fever control activities, with support from development partners including the Global Alliance for Vaccines and Immunization (GAVI Alliance), WHO and the United Nations Children Fund (UNICEF). The control activities involve the prevention of yellow fever outbreaks (through routine vaccination and regular preventive mass vaccination campaigns) and case-based yellow fever surveillance with laboratory confirmation and rapid mass vaccination response when an outbreak occurs [20].

The achievement and maintenance of high yellow fever vaccination coverage among infants through routine health services is an essential component of the plan. In 2004, routine yellow fever vaccination with one dose of the 17D yellow fever vaccine at nine months of age (the same age as the measles vaccine) was introduced in the whole country under the Expanded Programme on Immunisation (EPI). Routine childhood immunisation is offered in both public and private health centres in the country. Each health centre records vaccination acts in childhood vaccination cards and vaccination registers. At the beginning of each month, health centres compile the number of children who have received vaccines (including the yellow fever vaccine) and send the synthesis to the lead health centre in the health area. These synthesise the monthly vaccination acts for the health area and forward the reports to the district health service by the fifth day of each month. The district service makes a synthesis of vaccinations conducted in the health district and forwards this to the provincial level by the tenth day of each month, together with copies of the health area reports. The provincial level in turn makes its own synthesis and sends this to the Central Technical Group of the EPI by the fifteenth day of each month, together with copies of district syntheses that were used to compile the provincial report. The central level then makes a synthesis of district, provincial and national coverage. Each higher level sends feedback on performance to the lower level that provided it with data. The performance of routine yellow fever immunisation is assessed by the yellow fever vaccination coverage achieved and the coverage gap between the yellow fever and measles vaccine. While childhood immunisation is considered the long-term strategy for yellow fever control, several decades are needed to reduce susceptibility in all age groups. Therefore, to rapidly reduce susceptibility and prevent outbreaks in the meantime, yellow fever control activities in Cameroon include preventive mass vaccination campaigns in high-risk health districts. During the training of both vaccinators and supervisors for the campaigns, the procedures, tools and importance of data management during a mass vaccination campaign were explained. During the campaigns, the head of health area synthesises data from vaccination posts under their jurisdiction daily, identify problems and take appropriate action, and forward the syntheses to the district health service. The district makes its own synthesis and sends this to the provincial level, which in turn makes its own synthesis and forwards this to the central level. The central command post makes a daily synthesis of the campaign coverage and sends this feedback to the provincial level, which in turn sends feedback to the districts. At the end of a campaign, each province holds an evaluation meeting during which a final synthesis of district vaccination coverage is performed and transmitted to the central level.

We used WHO guidance documents [21,22] to develop written procedures for yellow fever surveillance, including case definition, case investigation forms, and instructions for specimen collection and transport, and outbreak investigation and response. This guidance material served as the basis for large-scale training in 2002 of central, provincial, district and health centre personnel using a snowball approach. These training sessions were also used as refresher courses for surveillance of other EPI diseases (acute flaccid paralysis [poliomyelitis], measles and neonatal tetanus). Following the training, case-based surveillance of suspected yellow fever cases was initiated in the country in January 2003. Since then, basic demographic and clinical information as well as one venous blood specimen are obtained from each suspected yellow fever case (i.e. a person who presents with fever followed by jaundice within two weeks of onset of fever). The blood specimen is well centrifuged and the resulting serum sent to the laboratory within three days of collection. Each blood specimen is tested for yellow-fever-specific IgM antibodies by use of rapid IgM enzyme-linked immuno-assay kits. A confirmed yellow fever case is defined as a suspected case with either laboratory confirmation via the IgM assay or epidemiological linkage to a laboratory-confirmed case. Currently, one confirmed case of yellow fever is considered an outbreak. Three key indicators are used to assess yellow fever case-based surveillance, i.e. the proportion of reported yellow fever cases with a blood specimen, the proportion of districts with at least one blood specimen in a year and the proportion of yellow fever outbreaks investigated with district outbreak reports sent to the central level. The target value for each indicator is a proportion of at least 80%.

Regular supportive supervision and meetings are used to monitor and improve the quality of data collection, analysis and reporting during both routine and supplementary activities. We have analysed the yellow fever vaccination and surveillance data reported to the EPI Central Technical Group in the Ministry of Public Health in Yaoundé from 2003 to 2006, and discussed the sustainability of yellow fever control activities in the country.

## Results

**Figure 1:**
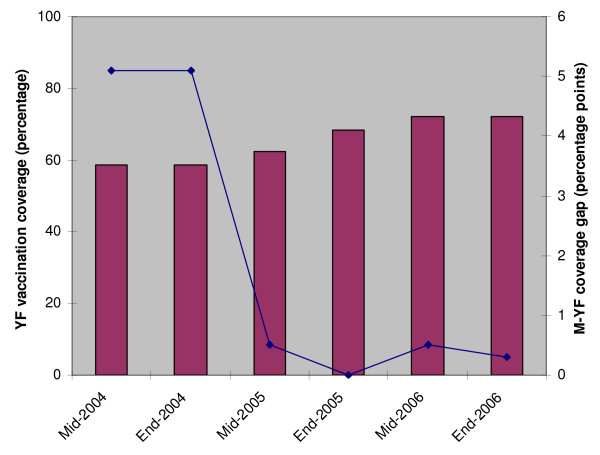
**Routine childhood vaccination coverage for yellow fever vaccine and the coverage gap for measles and yellow fever vaccines in Cameroon, 2004–2006**. M, measles; YF, yellow fever.

**Figure 2:**
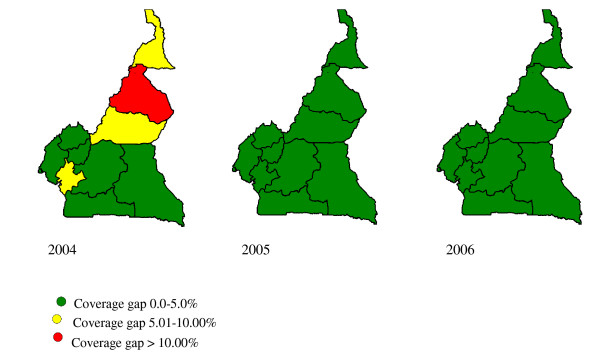
Maps of Cameroon showing the coverage gap between routine yellow fever and measles vaccination at sub-national level, 2004–2006.

Figure 1 shows the national yellow fever vaccination coverage and the coverage gap for yellow fever and measles vaccines in Cameroon from 2004 to 2006. Routine childhood yellow fever vaccination coverage rose from 58.7% in 2004 to 72.2% in 2006. Yellow fever vaccination coverage lagged behind measles vaccination coverage by more than five percentage points in 2004. From this timid start, the country rapidly achieved parity between yellow fever and measles vaccination coverage (i.e. not more than five percentage point difference between yellow fever and measles vaccination coverage) in 2005 and has since maintained this performance level. In addition, there was wide variation in yellow fever vaccination coverage levels across the country in 2004 (Figure 2) with only 6 out of 10 provinces achieving parity in yellow fever and measles vaccination coverage. However, yellow fever vaccination coverage levels have since improved with all 10 provinces achieving parity between yellow fever and measles vaccines since 2005 (Figure 2).

**Figure 3:**
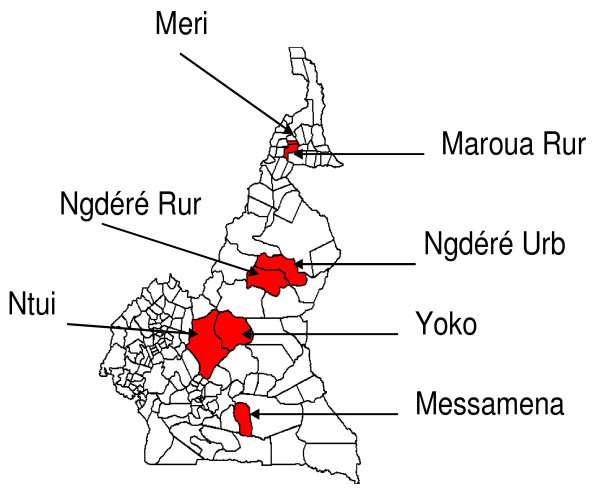
**Districts that conducted mass yellow fever vaccination campaigns in Cameroon in November 2006**. Ngdéré, Ngaoundéré; Rur, Rural; Urb, Urban.

In 2004, 16 high-risk districts with an estimated population of 1,848,158 people aged six months and above were selected for mass preventive vaccination campaigns. Each of the 16 districts was considered high risk either because it had reported a yellow fever outbreak within the last two decades or it has a large urban population in close proximity to a district that has recently reported an outbreak. The first phase of the preventive campaigns took place from 13 to 22 November 2006 in six districts (Figure 3), with vaccines supplied free from the GAVI Alliance yellow fever vaccine stockpile. During these supplementary immunisation activities, particular emphasis was placed on maintaining the cold chain, ensuring safe injection practices and surveillance of adverse events following immunisation (AEFIs). Vaccinators and supervisors were trained to recognise and report all adverse events in vaccinated persons following immunisation, and forms for reporting these AEFIs were distributed to all vaccination posts and health centres. During the 10 days of the vaccination campaigns, 752,195 (92.8%) people were vaccinated by 247 vaccination teams. The wastage rate for vaccines was 4.6% and that for safety boxes was 0.9%. Medical incidents were reported in 13 persons who had received the yellow fever vaccine (nine cases of injection site pain and swelling in the Ngaoundéré Urban health district and four cases of skin rash in the Yoko health district). None of these AEFIs was serious (i.e. life-threatening or required hospitalisation) and each was well managed by local health staff. There was high-level political commitment to these preventive vaccination campaigns which were officially launched in the Messamena health district of the East Province by the national Minister of Public Health, in the presence of WHO and UNICEF country representatives and high-ranking administrative and political leaders of the province. Following the successful implementation of the supplementary immunisation activities in the six districts, Cameroon plans to re-assess the risk of yellow fever outbreaks in the country [23] in 2007 in order to update the list of high-risk districts for the second phase of the preventive supplementary immunisation activities scheduled for 2008.

Table 1 shows the performance of yellow fever case-based surveillance in Cameroon. Since 2003 yellow fever surveillance in the country has been fully integrated with surveillance activities for acute flaccid paralysis and measles [16,18,24]. The surveillance of all three vaccine-preventable diseases share the same personnel and infrastructure at all levels of Cameroon's health care system, including personnel for reporting and investigation of suspected cases, specimen transport, and data management systems. The proportion of districts with at least one suspected yellow fever case per year increased from 31.4% in 2003 to 68.2% in 2006. Blood specimens were taken from all suspected yellow fever cases (within 14 days of onset of symptoms) and transported to and tested for yellow fever IgM antibodies at the Centre Pasteur in Yaoundé, Cameroon. All blood specimens that tested positive for IgM antibodies at the Centre Pasteur in Yaoundé were sent to the Pasteur Institute in Dakar, Senegal, for confirmatory tests. Cases of yellow fever were confirmed in Bafia, Méri and Ntui in 2003, Ngaoundéré Rural in 2004, Yoko in 2005 and Messamena in 2006 (Table 1). Reactive mass vaccination campaigns were conducted in Bafia (in January 2004) and Méri (in April 2004) health districts several months after confirmation of the yellow fever cases owing to constraints in mobilising the necessary resources. For the same reasons, vaccination campaigns were not organised in response to the epidemics in Ntui, Ngaoundéré Rural and Yoko health districts. However, these health districts were among the districts that conducted preventive mass vaccination campaigns in November 2006. The mass vaccination campaign in response to the epidemic in Messamena health district was also integrated into the preventive vaccination campaigns in high-risk districts in November 2006. The vaccination coverage was 93.8% (25,425/27,103) in Bafia and 84.5% (34,658/41,000) in Meri. In both districts, the mean wastage rates for vaccines and injection material were less than 5% and there were no reports of AEFIs.

**Table 1:** Yellow fever case-based surveillance in Cameroon, 2003–2006

**Year**	**Number of districts**	**Districts reporting at least one suspected case**		**Number of suspected cases reported**	**Blood specimens collected**		**Confirmed cases**		**Districts with confirmed cases**
		
	***n***	*** n ***	** % **	***n***	*** n ***	** % **	*** n ***	** % **	
2003	144	52	36.1	156	156	100	3	1.3	Bafia, Méri, Ntui
2004	159	80	50.3	434	434	100	2	0.5	Ngaoundéré Rural
2005	159	88	55.3	831	831	100	2	0.2	Yoko
2006	167	114	68.2	859	859	100	1	0.2	Messamena

## Discussion

The government of Cameroon, with assistance from her development partners, has since 2003 been implementing strategies both to prevent yellow fever outbreaks and to detect and respond appropriately to the outbreaks when they occur. Routine yellow fever vaccination coverage has improved dramatically with the country achieving parity between yellow fever and measles vaccination coverage within a year of introducing the yellow fever vaccine into the routine childhood immunization schedule. In addition, districts at high risk of yellow fever outbreaks were identified for preventive supplementary immunisation activities. The first phase of the latter was very successful in terms of vaccination coverage (93%) and quality (i.e. no serious AEFI was reported). In addition, the case-based yellow fever surveillance system is being used to update the list of high-risk districts. However, the major constraint has been responding promptly to a declared yellow fever outbreak. Ideally, a mass vaccination campaign should be organised within days of confirming a yellow fever epidemic in order to contain the outbreak. This has not been the case, owing to difficulties in rapidly mobilising resources amidst other competing health priorities. However, Cameroon has made remarkable preliminary progress in controlling yellow fever in recent years. The challenge now is to complete the planned preventive mass vaccination campaigns within schedule and to increase and maintain high routine vaccination coverage. In addition, measures have to be taken to improve and maintain high-quality case-based surveillance in all health districts, promptly plan and conduct reactive mass campaigns when outbreaks occur and continuously monitor AEFIs.

Even though our team and other investigators in Africa [25] did not find serious adverse events following yellow fever immunisation, AEFIs have recently become a big issue in South America and non-endemic countries where travellers are immunized against yellow fever [26,27]. In the early years of yellow fever vaccine production, post-vaccination encephalitis was common but such events were markedly reduced after the adoption of seed lot manufacturing in 1941 and the restriction of vaccination to persons older than six months of age after 1969 [27,28]. Vaccine-associated neurotropic disease is currently estimated to occur at a frequency of 4 per 1,000,000 doses [29]. Within the past decade, a more severe adverse event following yellow fever immunisation emerged [30-32]. In 2001, seven cases of severe acute viscerotropic disease following immunisation were reported; six of these were fatal [30-32]. This condition mimics severe yellow fever and is estimated to occur at a frequency of 3 per 1,000,000 doses [29]. The mechanism underlying vaccine-associated neurotropic or viscerotropic events is poorly understood. These events could be a result of vaccine virus reversion (or mutation) to wild-type virulence or a result of host-specific factors [27,28]. Whatever the underlying cause, each AEFI requires a thorough investigation to establish whether it is caused by the vaccine itself, by an error in the administration of the vaccine or not related to the vaccine or administration (i.e. its occurrence merely coincided with vaccine administration). AEFIs may upset the public and lead to refusal of further immunisation. If this were allowed to occur, hundreds of thousands of people will continue to contract yellow fever each year and tens of thousands of these will die. Therefore, surveillance of AEFIs helps to preserve public confidence in immunisation programmes and should be an integral part of all yellow fever immunisation programmes.

Opportunities for improving yellow fever control in Cameroon include the recently formed Yellow Fever Initiative [10] and the high-level political commitment to the control of vaccine-preventable diseases in the country [16,19]. The Yellow Fever Initiative will assist 12 priority countries in West Africa, including Cameroon, to conduct mass preventive vaccination campaigns targeting 48 million people between 2007 and 2010. These preventive campaigns should dramatically reduce the yellow fever disease burden in these countries, just as similar campaigns did for measles [33-35]. Later in 2007, Cameroon will conduct a yellow fever risk analysis using the WHO decision-making tool for preventive immunization campaigns [23] in order to update the list of high-risk districts for preventive mass campaigns next year.

The long-term strategy for yellow fever control in the country, as elsewhere, remains high routine childhood vaccination coverage. High vaccination coverage should be attained and maintained among infants, before their first birthday, in every district. The operational strategies for achieving and sustaining high routine vaccination coverage (90% or more) in each successive birth cohort include fostering high-level political commitment, improving the planning and monitoring of immunisation services at all levels, community mobilisation, efficient coordination of current immunisation partners and mobilisation of additional funding for immunisation services.

The EPI was restructured in 2002 and is now a prominent organisational structure in the Ministry of Public Health, and the government is committed to sustaining routine vaccination services by making vaccines and vaccination material (vaccination cards, vaccination registers, syringes and safety boxes) permanently available at all levels of the health system. The restructured programme focuses maximum financial and technical support at the district level. Districts with low vaccination coverage are identified and given technical assistance in planning and monitoring vaccination activities. Emphasis is placed on supportive supervision, regular meetings to discuss data, use of data for programmatic action and regular feedback. Novel operational strategies developed at district and health facility levels include keeping log books in all villages for a census of all newborns and conducting special immunisation days in hard-to-reach areas, those without health units and or health workers and those with low vaccination coverage. Since vaccination coverage is strongly associated with socio-economic status in Cameroon [36], communication and social mobilisation efforts that accompany and support vaccination delivery have been adapted to each milieu. Permanent community dialogue structures have been established in all health districts and efforts are made to reach poorly educated mothers by disseminating information through women's associations and community opinion leaders, resulting in increased demand for vaccination services [19].

## Conclusion

In conclusion, amidst other competing health priorities, Cameroon is using experience gained from controlling other vaccine-preventable diseases to plan and implement evidence-based strategies for preventing yellow fever outbreaks and for detecting and responding to outbreaks when they occur.

## Competing interests

The author(s) declare that they have no competing interests.

## Authors' contributions

All authors participated in various components of the yellow fever control programme in Cameroon which is coordinated by EN. CSW conceived the paper and wrote the first draft. All authors contributed important intellectual content to the article, and read and approved the final version.

## Pre-publication history

The pre-publication history for this paper can be accessed here:


